# Supplementary material for: The effects of psychological flexibility and night shifts on mental health and well-being in nurses
Source: PLoS One. 2024 Nov 14;19(11):e0313634. doi: 10.1371/journal.pone.0313634 (PMC11563484; doi:10.1371/journal.pone.0313634)
Supplement: Attachment — Submitted filename: Response to comments.docx [file pone.0313634.s001.docx]

Response to comments:

Editor comments:

1. Abstract. The Results section is a bit reiterative. The same information is given with and without numerical data. Try to sum up the information.

Response: I edited: lines 40-46.

2. Abstract. Conclusions are very short and should give more information on night shifts.

Response: I edited: lines 47-51.

3. Introduction. Increase the literature evidence about night shifts. The information about night shifts and its health-related implications should be enhaced. Also, the IARC puts night shifts as predictor of cancer. Night shifts have implications on work-family conflicts and changes in lifestyle too. Examples can be found here: https://pubmed.ncbi.nlm.nih.gov/33321692/ ; https://pubmed.ncbi.nlm.nih.gov/33806956/ ; <https://pubmed.ncbi.nlm.nih.gov/34070908/>.

Response: I added: lines 105-109

4. Methods. Increase the information about the study procedure. Did all nurses worked in the same clinical areas or there were differences (emergency, surgery, hospitalization, infectious diseases...)? Was it a written or virtual-based survey? If written, how did you computerized the responses? What program did you use to compile information before applying AMOS? Include information about anonymization of the responses.

Response: I added: lines 121-122, 128, 136-139,

5. Results. Information resulting from Table 4 is confusing (difficult to read), I recommend to check the paragraph about direct and indirect effects.

Response: I edited: lines 190-197, 215-230.

6. Discussion. Information about night shifts is general, more appropriate for the Introduction. Whereas, authors should increase relations between night shifts and mental health, psychological well-being.

Response: I added: lines 287-290, 295-303.

7. Limitations. You mention the number of questions, how many questions were in the questionnaire? how much time did it take to complete the survey? Did participants refused to complete the survey because they were tired? I do not see information about nurses areas of work (emergency, surgery, hospitalization...), so there might be important differences depending on the area that the authors did not consider.

Response: I removed this limitation, because I had not measured it. I added new limitations. Lines: 309-328.

8. Conclusions. The conclusions are vague, and some numerical data and principal variables should be mentioned.

Response: I edited: lines: 330-342.

**Reviewer #1:**

Dear authors,
I have read your paper with great interest. The paper is rich in information that provides valuable information in the field of nursing. This study aims to assess the mental health and well-being of Chinese nurses, as well as investigate the impact of psychological flexibility and night shifts on this relationship. I believe implementing the following suggestions will enhance the overall quality of your paper:
Title:
Please consider revising the title to make no more than 15 words.

Response: The journal has not set a limit on the number of tile words. However, with the approval of the editor, I can change this title to: ‘’The effects of psychological flexibility and night shifts on mental health and well-being in nurses’’
Abstract:
the 10 Grade II public hospitals. I think this information is not necessary. Please add the statistical issues in your abstract.

Response: The place of data collection was these hospitals, so removing it from the methods cannot be correct. However, I removed the number and wrote the detailed section on data collection in hospitals. Briefly, I also added analysis tools. Lines 33, 38-39.
Conclusion:
social support in the workplace helps young nurses cope better with job stress and protect their mental health and wellbeing. How you conclude that since your study does not investigate social support. Please re-write your conclusion to be consistent with your findings.

Response: I edited it: lines 47-51.
Keywords: add flexibility word.

Response: I added in keywords.
Introduction:
The introduction is well-written. However, there are some missing paragraphs for demographic factors included in your study. Why they are important and what the literature says about them. I highly recommend before the method section to write the significant values of your study and make a good connection between your variables.

Response: I added: lines 110-115.

Method:
-public hospitals: how many?

Response: 10 Grade II public hospitals in Shanghai: line:125.
-Clarify the data time collection period.

Response: September 20 to December 20, 2023: line: 121.
-Address the issue of data collection such as how many nurses respond and what is the response rate. (Maybe as an appendix Table).

Response: I added it in results section: lines: 184-187.
Add a reference for the normal values of CFI, TLI, REMSA…etc in your analysis section.

Response: I added: lines 210-211.
Results
Appropriate.
Discussion:
-Please highlight your findings instead of repeating your aims.
-being more psychologically flexible. Please how nurses can be??? Write more details in application.
-write more details regarding the connection between your variables.
- I noticed that the majority of references were from China. I respect that. But I suggest strengthening your paper by adding more international studies regarding your topic.

Response: I added some texts in this section.
Finally, I hope to add clinical implications for nurses to reduce the effect of the night shift on their lives. Some nurses are obligated to work in night shift for several reasons (study, home). Please take this into your mind.

Response: I edited limitation: lines: 326-335.
References:
Please double-check according to the journal style.

Response: I edited it.
Tables:
Ensure that abbreviations used in tables are mentioned in the footnotes.

Response: yes, thanks.

**Reviewer #2:**

Thank you for reviewing of this paper. The manuscript is well written in standard English. The statistical Analysis method should be somewhat clear. The recommendation should be revised and rewritten based on your findings.

Response: I edited

Lines: 326-335.
