# Supplementary material for: The effects of psychological flexibility and night shifts on mental health and well-being in nurses
Source: PLoS One. 2024 Nov 14;19(11):e0313634. doi: 10.1371/journal.pone.0313634 (PMC11563484; doi:10.1371/journal.pone.0313634)
Supplement: Attachment — Submitted filename: Response to comments.docx [file pone.0313634.s002.docx]

**Reviewer #1: Dear Authors,**

**I have noticed that my comments were not taken into consideration, especially in the Methods and Discussion sections. It is important that these issues are addressed thoroughly to enhance the quality and clarity of your manuscript.**

**Best regards.**

**Response: Thanks to the first reviewer: I have reviewed your previous comments and revised the text accordingly.**

**Previous comments of the Reviewer #1:** 
Title:
Comment: Please consider revising the title to make no more than 15 words.

Response: I changed the title to: The effects of psychological flexibility and night shifts on mental health and well-being in nurses----- lines: 1-2

Abstract:
Comment: the 10 Grade II public hospitals. I think this information is not necessary. Please add the statistical issues in your abstract.

Response: I edited it; lines 32-40.

Conclusion:
Comment: social support in the workplace helps young nurses cope better with job stress and protect their mental health and wellbeing. How you conclude that since your study does not investigate social support. Please re-write your conclusion to be consistent with your findings.

Response: I edited it: lines 48-52.

Comment: Keywords: add flexibility word.

Response: I added in keywords. Line: 53.

Introduction:
Comment: The introduction is well-written. However, there are some missing paragraphs for demographic factors included in your study. Why they are important and what the literature says about them. I highly recommend before the method section to write the significant values of your study and make a good connection between your variables.

Response: I added some demographic factors in lines 80-88 and 115-119. Also, in lines 125-130, I added the significant values of study.

Method:
Comment: public hospitals: how many?

Response: 10 Grade II public hospitals in Shanghai were selected.: line:140.

Comment: Clarify the data time collection period.

Response: September 20 to December 20, 2023: line: 136.

Comment: Address the issue of data collection such as how many nurses respond and what is the response rate.

Response: I added it in results section: In general, 612 questionnaires were distributed among nurses. 564 questionnaires were filled by the participants (the response rate was equal to 92.15%). However, only 422 questionnaires were approved (the correct response rate was equal to 74.82%) (27.34 ±4.19 years) and the rest of the questionnaires were not completely filled and therefore were excluded from the study. lines: 201-205.

Comment: Add a reference for the normal values of CFI, TLI, REMSA…etc in your analysis section.

Response: I added reference in statistical section. GFI >0.95 [37], NFI >0.95 [38], CFI >0.96 [39], AGFI >0.90 [37], RMSEA <0.05 [37], and TLI >0.90 [37] indicated the fit of the model. Lines: 195-196.

Results
Appropriate.

Discussion:

Comment: being more psychologically flexible. Please how nurses can be??? Write more details in application.

Response: I added some contents about it in discussion section: lines 318-325. (In general, it can be said that psychological flexibility was one of the factors affecting the mental health and well-being of nurses, which was lower in male nurses and younger nurses. Studies show that one of the ways to increase psychological flexibility is the use of Acceptance and Commitment Therapy (ACT) interventions [62, 63]. ACT focuses on pursuing important life areas and goals, like close relationships, fulfilling work, and personal development, even when facing difficult experiences. In ACT, this is achieved by developing psychological flexibility, which helps people stay involved in meaningful activities, even when they have negative thoughts, feelings, or face other challenges [63]).

Comment: write more details regarding the connection between your variables.

Response: I edited whole of discussion section.

Comment: I noticed that the majority of references were from China. I respect that. But I suggest strengthening your paper by adding more international studies regarding your topic.

Response: I added some studies from other studies in introduction and discussion sections (Studies from Australia, UK, Jordan, Vietnam, and...).

Comment: Finally, I hope to add clinical implications for nurses to reduce the effect of the night shift on their lives. Some nurses are obligated to work in night shift for several reasons (study, home). Please take this into your mind.

Response: I added it: lines: 352-362.

References:
Comment: Please double-check according to the journal style.

Response: I have used Endnote software for writing references. I used the PloS reference writing style.

**Reviewer #2:**

Reviewer #2: Thank you for inviting me to review this paper. The title “The effects of psychological flexibility and night shifts on the relationship between mental health and well-being in Chinese nurses: A systematic review” was very interesting.

My concerns are mentioned below:

Keywords; mental health, nurse, night shift, wellbeing, psychological flexibility make the first letter of each word capitalize.

Overall the paper is well modified

Response: I edited it.
